# Supplementary material for: Phosphorylation-deficient G-protein-biased μ-opioid receptors improve analgesia and diminish tolerance but worsen opioid side effects
Source: Nat Commun. 2019 Jan 21;10:367. doi: 10.1038/s41467-018-08162-1 (PMC6341117; doi:10.1038/s41467-018-08162-1)
Supplement: Supplementary file 1 — Supplementary Information [file 41467_2018_8162_MOESM1_ESM.docx]

**Phosphorylation-deficient G-protein-biased μ-opioid receptors improve analgesia and diminish tolerance but worsen opioid side effects**

*A. Kliewer^1^, F. Schmiedel^1^, S. Sianati^2^, A. Bailey^3^, J. T. Bateman^4^, E. S. Levitt^4^, J. T. Williams^5^, M. J. Christie^2^ and S. Schulz^1*^*

# Supplementary Methods

**Western blot analysis**

Mice were anesthetized with isoflurane, killed by cervical dislocation, and brains were quickly dissected excluding the cerebellum. The remaining brain samples were immediately frozen in liquid nitrogen. Brains were transferred to ice-cold detergent buffer (50 mM Tris-HCl, pH 7.4, 150 mM NaCl, 5 mM EDTA, 10 mM NaF, 10 mM disodium pyrophosphate, 1% Nonidet P-40, 0.5% sodium deoxycholate, 0.1% sodium dodecyl sulfate (SDS), containing protease and phosphatase inhibitors), homogenized, and centrifuged at 14,000 × *g* for 30 min at 4°C. The supernatant was added with SDS-sample buffer, incubated for 5 min at 95°C and then resolved on 8% SDS-polyacrylamide gels. After electroblotting, membranes were incubated with the anti-β-arrestin1 (ab32095, abcam) or anti-GRK2 (sc-562, Santa Cruz Biotechnology) antisera followed by detection using an enhanced chemiluminescence detection system (Amersham, Braunschweig, Germany). Blots were subsequently stripped and reprobed with anti-actin (sc-47778, Santa Cruz Biotechnology) to confirm equal loading of the gels. Protein bands on Western blots were exposed to X-ray film. Films exposed in the linear range were then densitized using ImageJ 1.37v.

## Brain slice electrophysiology recordings of Kölliker-Fuse neurons

## Coronal (230 µm) slices containing Kölliker-Fuse (KF) neurons were prepared as described previously^21^. Whole-cell recordings were made from KF neurons with a Multiclamp 700B amplifier in voltage-clamp mode (V_hold_ = -60 mV). Recording pipettes (1.5 – 2.2 MΩ) were filled with internal solution containing (in mM) 115 potassium methanesulfonate, 20 NaCl, 1.5 MgCl_2_, 5 HEPES(K), 2 BAPTA, 2 Mg-ATP, and 0.2 Na-GTP, pH 7.4, 275-280 mOsM. Data were filtered at 10 kHz and collected at 20 kHz with pClamp10 or 400 Hz with PowerLab (Chart version 5.4.2; AD Instruments, Colorado Springs, CO). Series resistance was monitored without compensation and remained < 15 MΩ for inclusion. KF neurons with an opioid-mediated outward current (~60 % of KF neurons) fired action potentials at a slower frequency after injection of current steps (50 – 250 pA, 1 s) and had a smaller amplitude AHP^21^. Drugs (including [Met^5^]enkephalin (ME), baclofen, CGP55845 and idazoxan) were applied by bath perfusion at the indicated concentrations. Bestatin (10 µM) and thiorphan (1 µM) were included with ME to prevent degradation.

**Conditioned place preference test (CPP)**

The rewarding properties of opioids were measured in a 3-chambered CPP box (TSE Systems, Bad Homburg, Germany). Each of the three compartments was distinct. In the center compartment all walls and floor were light grey. One of the two “choice” compartment was white with a ribbed floor whereas the other compartment was striped black-and-white with a nub floor. The CPP procedure consisted of pre-conditioning (days 1-2, baseline on day 2), conditioning (days 3-8), and post-conditioning (day 9). On days 1-2 and day 9, mice were allowed free access to all 3 chambers for 15 min. On day 2 (baseline), the amount of time mice spent on each side (left or right) during a 15-min testing period was recorded using a TSE Videomot system (TSE Systems, Bad Homburg, DE). Only mice with no significant place preference to one of the two compartments during pre-conditioning period underwent subsequent conditioning (unbiased) and were additionally drug conditioned to the less-preferred chamber ^42, 45^. During conditioning, the less-preferred compartment was paired to drug injection while the other compartment to saline injections. The conditioning phase (15 min per session with fentanyl and 30 min per session with morphine) was carried out daily over 6 consecutive days with alternate injections of drug or saline. WT and 11S/T-A mice were exposed to either fentanyl (0.1 mg kg^-1^) or morphine (7.5 mg kg^-1^) on days 3, 5 and 7 and saline on days 4, 6 and 8. On day 9, the mice were given free access to all 3 chambers for 15 min and time spent on each side (left or right) was recorded. We repeated the experiment two times for both fentanyl and morphine with 6 mice per genotype per experiment (N=12). CPP was calculated (Δ) as (time spent in the drug-paired side post-conditioning) – (time spent in the drug-paired side pre-conditioning)^42, 45^. Data were analyzed using one-way ANOVA, followed by Bonferroni’s *post hoc* tests or two-tailed *t* test with significance set at P < 0.05.

**Supplementary Figure 1**


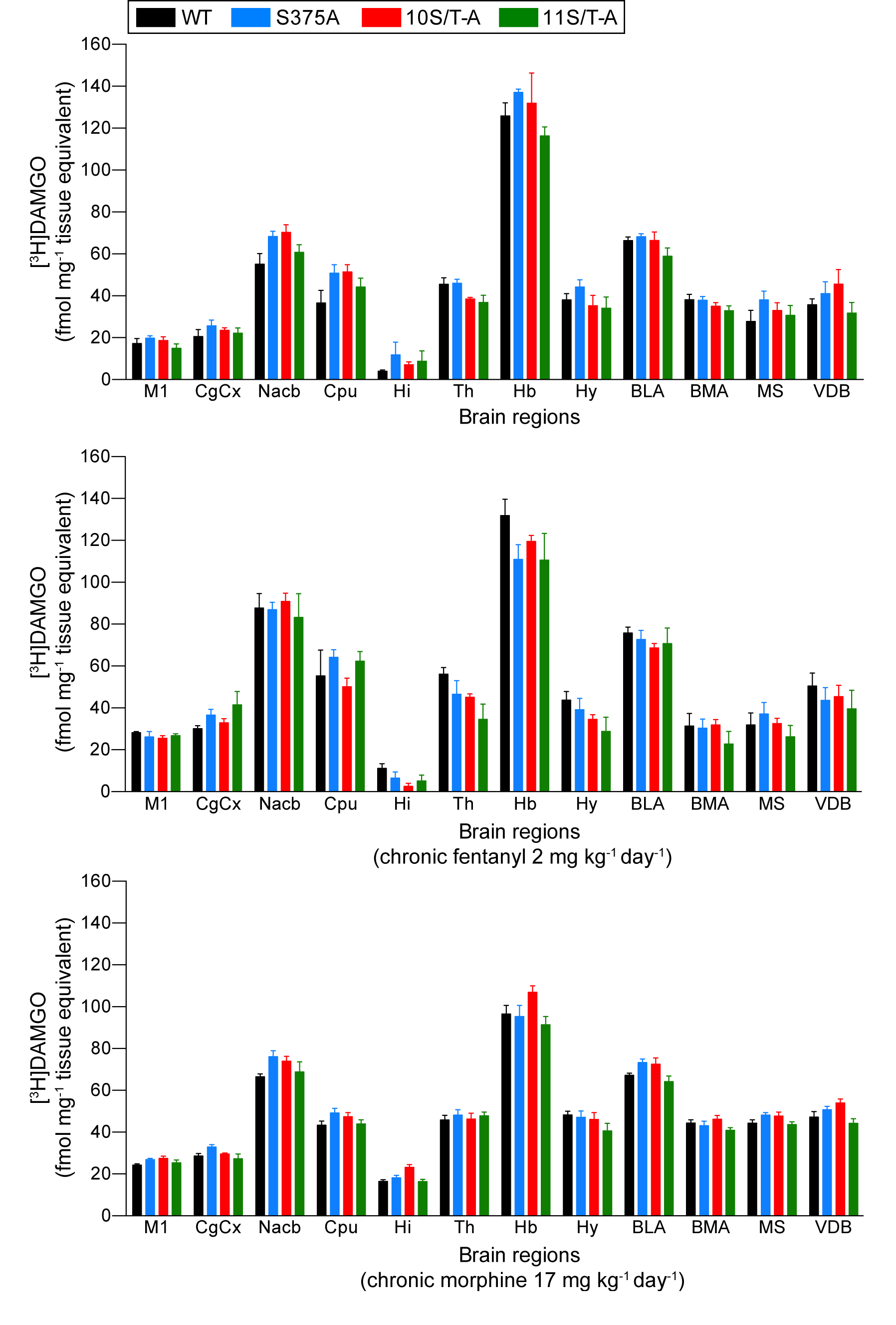


### Supplementary Figure 1 No significant alteration in MOP [^3^H]DAMGO binding in phosphorylation-deficient MOP knockin mice.

**a, b, c,** Qua**ntitative autoradiography of** [^3^H]DAMGO binding to MOP in coronal brain sections from **a,** naïve WT, S375A, 10S/T-A and 11S/T-A mice and after chronic treatment with osmotic pumps delivering **b**, fentanyl (2 mg kg^-1^ day^-1^) or **c,** morphine (17 mg kg^-1^ day^-1^) over 7 days. Data are expressed as the mean specific binding (fmol mg^-1^) ± s.e.m. (n = 3–6). The colour bar represents a pseudo-colour interpretation of black and white film images in fmol mg^-1^ tissue equivalent with nonspecific binding (NSB) at background level. Abbreviations: M1, Motor cortex; CgCx, Cingulate cortex; Nacb, Nucleus accumbens; CPu, Caudate putamen; Hi, hippocampus; Th, thalamus; Hb, habenula; Hy, Hypothalamus; BLA, basolateral amygdala; BMA, basomedial amygdala; MS, medial septum; VDB, vertical limb diagonal band. Two-way ANOVA with Bonferroni *post hoc* test.

**Supplementary Figure 2**

**
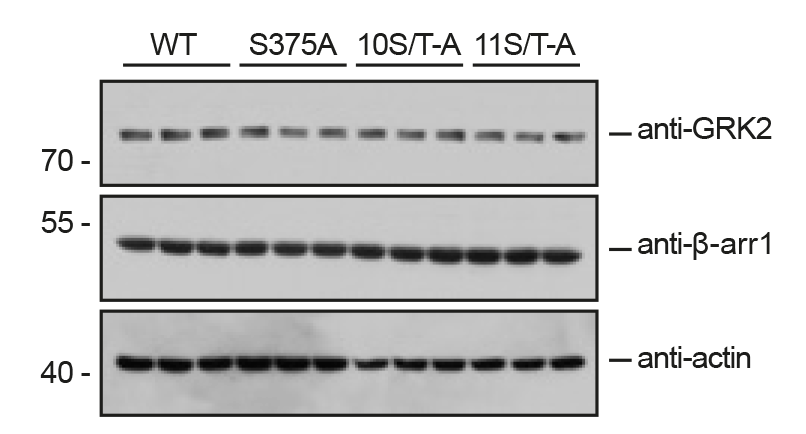
**

**Supplementary Figure 2 Western blot analysis of ß-arrestin1 and GRK2 in brain lysates.** Brain lysates from WT, S375A, MOR10S/T-A and MOR11S/T-A (n = 3) were analysed for expression of GRK2 and ß-arrestin1. Blots were stripped and probed with anti-actin antibody to confirm equal loading. The positions of molecular mass markers are indicated on the *left* (in kDa).

**Supplementary Figure 3**

**
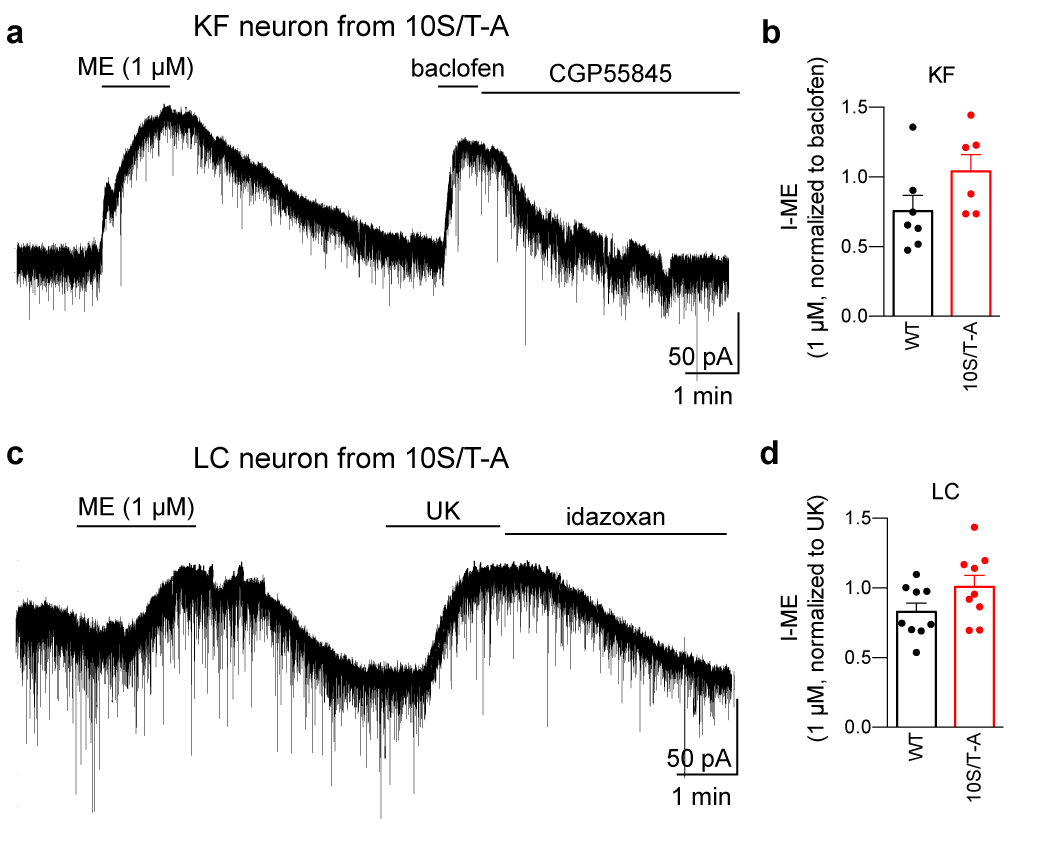
**

**Supplementary Figure 3 Retained opioid-activated GIRK conductance in the respiratory-related KF nucleus from 10S/T-A mice. a,** Representative whole-cell voltage-clamp recording from a Kölliker-Fuse (KF) neuron from a 10S/T-A mouse showing outward current mediated by [Met^5^]enkephalin (ME 1 µM) and baclofen (30 µM). The GABA-B antagonist CGP55845 (300 nM) was included in washout of baclofen. The current induced by ME was normalized to that induced by baclofen. **b,** Normalized ME-mediated currents were similar in KF neurons from wild-type and 10S/T-A mice (P = 0.11). **c,** Representative whole-cell voltage-clamp recording from a locus coeruleus (LC) neuron from a 10S/T-A mouse. The current induced by ME (1 µM) was normalized to that induced by UK14304 (3 µM). Idazoxan (1 µM) was included in washout of UK14304. **d,** Normalized ME-mediated currents were similar in LC neurons from wild-type (WT) and 10S/T-A mice (P = 0.10). Data are the means ± s.e.m.; unpaired t-test (n = 6–9).

**Supplementary Figure 4**

**
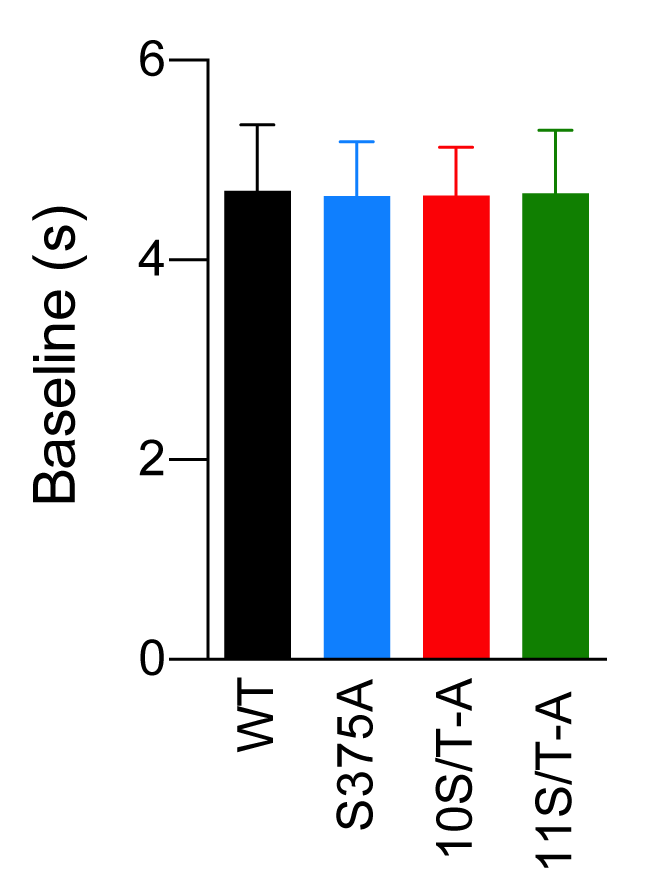
**

**Supplementary Figure 4 Basal pain responses did not differ among genotypes.** Basal pain responses determined at a hot-plate temperature of 56°C among different genotypes (F_(3, 86)_ = 0.04070, P = 0.9890) (n = 21–24). Data are the means ± s.e.m. One-way ANOVA with Bonferroni *post hoc* test.

## Supplementary Figure 5

**
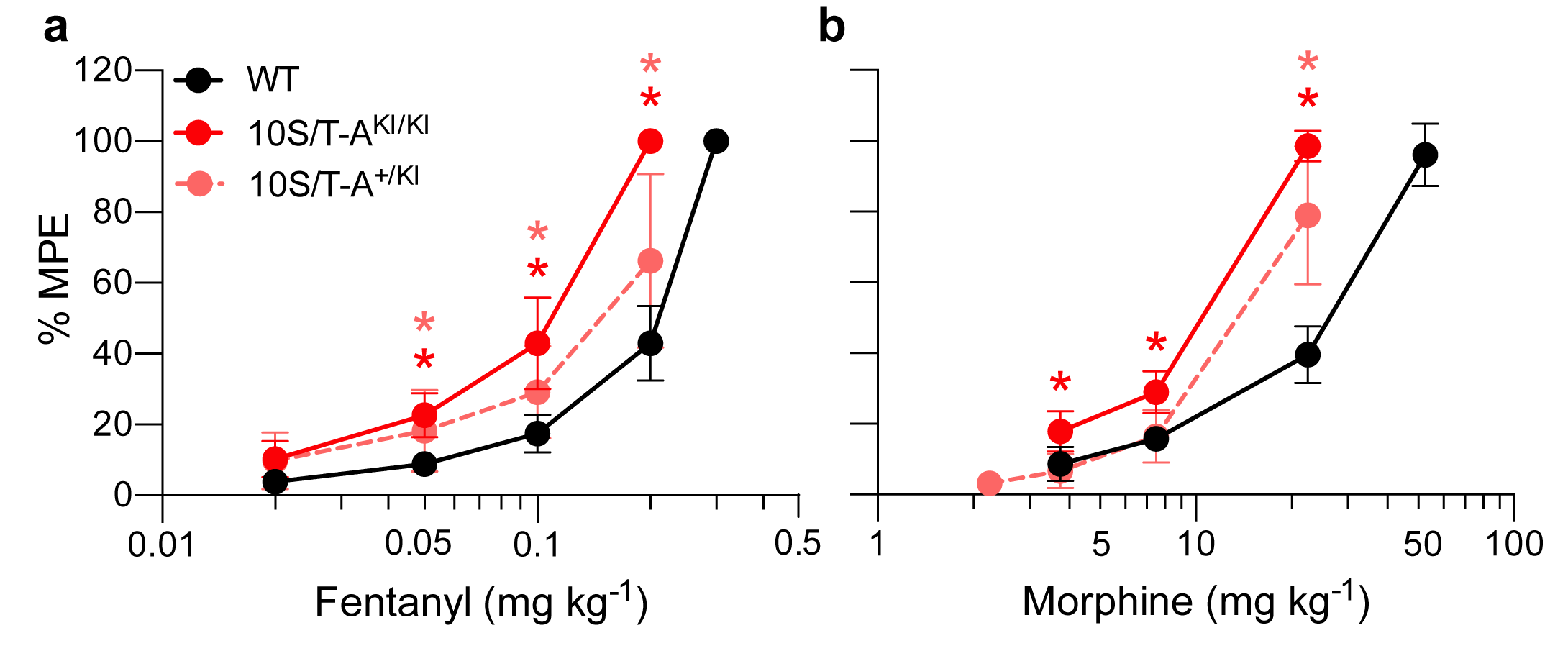
**

### Supplementary Figure 5 Heterozygous 10S/T-A mice also exhibited greater antinociception.

**a, b,** Acute antinociceptive response measured in the mouse hot-plate test. Nociceptive latencies were defined by paw withdrawal and are reported as percent maximum possible effect (% MPE) with a 30-s cut-off. Cumulative dose-response curves in 10S/T-A^KI/KI^ and 10S/T-A^+/KI^ compared to that in WT mice (n = 9–12) after **a,** fentanyl or **b,** morphine.

## Supplementary Figure 6

**
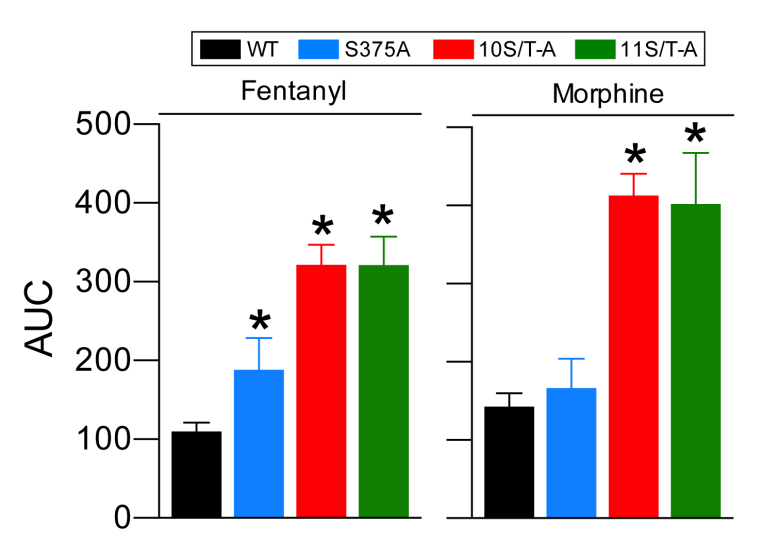
**

### Supplementary Figure 6 Area under the curve of analgesic time course.

Increased analgesic responses in 10S/T-A and 11S/T-A knockin mice compared to that in WT (n = 6–7) after acutely administered fentanyl (F_(3, 21)_ = 71.68, P < 0.0001) or morphine (F_(3, 21)_ = 73.22, P < 0.0001). Data are the means ± s.e.m.; * indicate statistically significant differences compared to WT. One-way ANOVA with Bonferroni *post hoc* test. AUC: area under the curve.

## Supplementary Figure 7

**
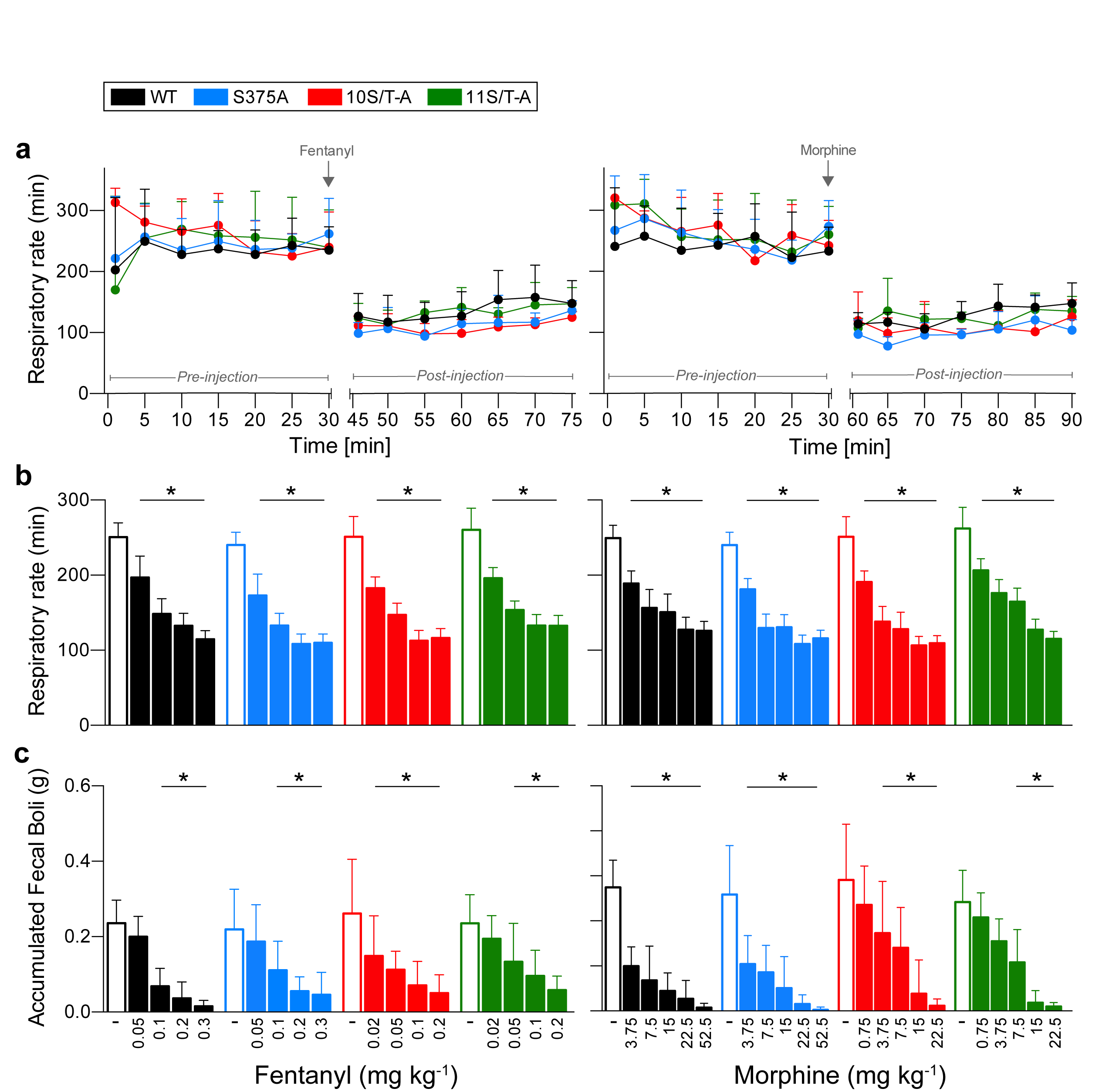
**

### Supplementary Figure 7 Respiratory depression and constipation dose-response curves.

**a,** Time course of respiratory rates measured by plethysmography 15 min (fentanyl) or 30 min (morphine) after administration of equally effective doses for analgesia of fentanyl (0.1 mg kg^-1^ for 10S/T-A and 11S/T-A; 0.2 mg kg^-1^ for WT and S375A) or morphine (15 mg kg^-1^ for 10S/T-A and 11S/T-A; 22.5 mg kg^-1^ for WT and S375A). **b, c,** Dose-response curves for calculated ED_50_ values. **b,** Respiratory rate measured by plethysmography 15 or 30 min after drug administration (fentanyl: F_(19, 15820)_ = 5033, P < 0.0001; morphine: F_(23, 19336)_ = 4985, P < 0.0001) (n = 6). **c,** Accumulated faecal boli weight in the constipation test (fentanyl: F_(19, 188)_ = 12.31, P < 0.0001; morphine: F_(23, 188)_ = 17.83, P < 0.0001) (n = 6–16). Data are the means ± s.e.m. * indicate statistically significant differences between drug and vehicle; one-way ANOVA with Bonferroni *post hoc* test.

**Supplementary Figure 8**


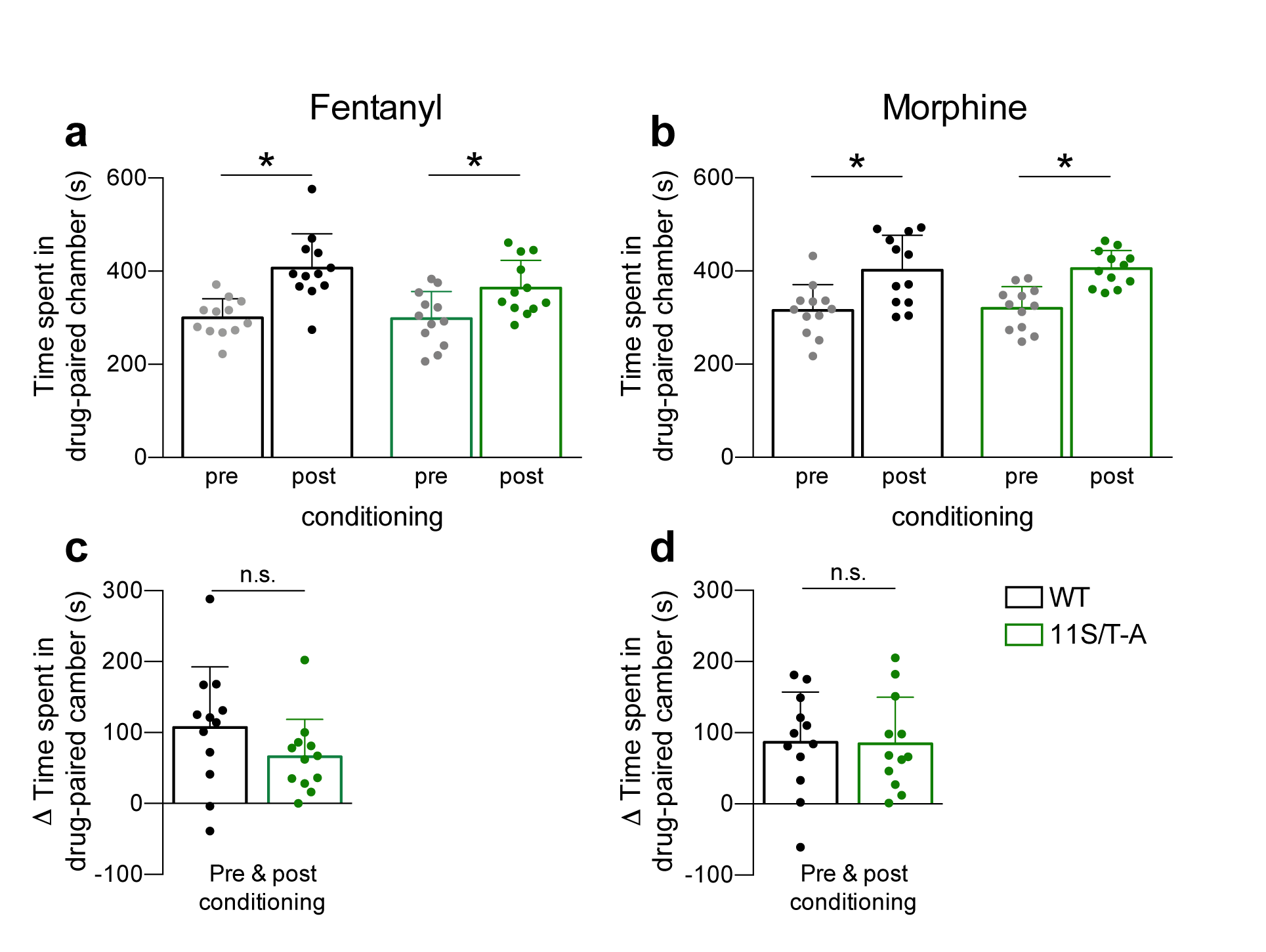


### Supplementary Figure 8 Retained opioid seeking behaviour.

**a-d,** On the pre-conditioning day, WT or 11S/T-A mice were assessed for time spent in one of the CPP compartments. Six days after the conditioning regime (0.1 mg kg^-1^ fentanyl or 7.5 mg kg^-1^ morphine on days 1, 3 and 5 and saline on days 2, 4 and 6) time spent in each CPP compartment was recorded again over 15 min (post-conditioning day). **a, b,** Pre- and post-conditioning time in drug-paired chamber of the conditioned place preference apparatus. Data are means ± s.e.m; * indicates statistically significant differences comparing pre- and post-conditioning time in the drug-paired chamber from each genotype (fentanyl: F_(3, 44)_ = 9.656, P < 0.0001 (n = 12) and morphine: F_(3, 44)_ = 9.529, P < 0.0001) (n = 12); one-way ANOVA with Bonferroni *post hoc* test. **c, d,** Data are shown as the difference (Δ) in time spent between the drug-paired compartment on the post-conditioning day and the pre-conditioning day. Data are means ± s.e.m; comparison of WT vs 11S/T-A in a two-tailed *t* test: fentanyl CPP (P = 0.0807) (n = 12) and morphine CPP (P = 0.9438) (n = 12).

## Supplementary Figure 9

**
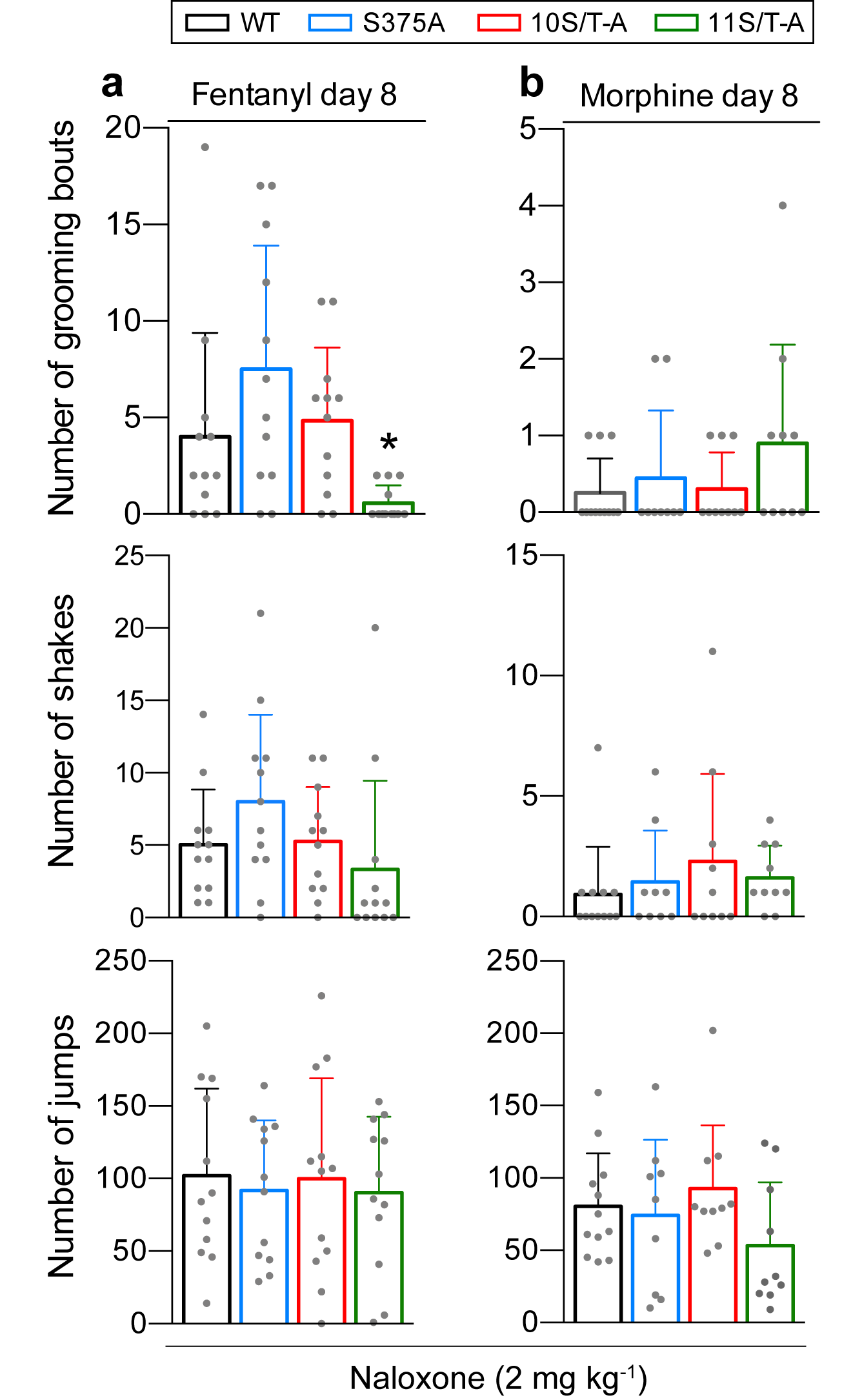
**

### Supplementary Figure 9 Retained signs of dependence.

**a, b** Naloxone-precipitated withdrawal signs on day 8 after chronic treatment with osmotic pumps delivering **a,** fentanyl (2 mg kg^-1^ day^-1^) or **b,** morphine (17 mg kg^-1^ day^-1^) (n = 9–12). Grooming bouts, shakes and jumps were measured after 2 mg kg^-1^ naloxone and calculated in the global withdrawal scores. Data are the means ± s.e.m; * indicates statistically significant differences compared to WT, unpaired, two-tailed *t* test.

## Supplementary Table 1 pED_50_ values for analgesia, respiration and constipation.

|  | Fentanyl (mg kg^-1^) | | | Morphine (mg kg^-1^) | | |
| --- | --- | --- | --- | --- | --- | --- |
|  | *Analgesia* | *Respiration* | *Constipation* | *Analgesia* | *Respiration* | *Constipation* |
| WT | 0.21 ± 0.005 | 0.09 ± 0.019 | 0.07 ± 0.023 | 26.64 ± 1.498 | 11.35 ± 1.856 | 13.10 ± 1.494 |
| S375A | 0.15 ± 0.008* | 0.08 ± 0.003 | 0.09 ± 0.009 | 25.09 ± 1.477 | 8.32 ± 2.454 | 11.57 ± 0.852 |
| 10S/T-A | 0.12 ± 0.004* | 0.04 ± 0.014* | 0.05 ± 0.013 | 12.72 ± 0.222* | 2.61 ± 1.667* | 8.17 ± 0.852* |
| 11S/T-A | 0.12 ± 0.006* | 0.03 ± 0.000* | 0.05 ± 0.024 | 11.10 ± 0.361* | 3.76 ± 2.454 | 7.05 ± 1.2* |

Summary of pED_50_ values (mg/kg) for cumulative analgesic dose–response curves measured in the hot-plate (day −1), constipation, and respiratory depression tests. Data are the means ± s.e.m. * indicate statistically significant difference compare pED_50_ values to WT (for fentanyl: F_analgesia(3, 44)_ = 56.47, P < 0.0001; F_respiration(3, 92)_ = 6.435, P = 0.0005; F_constipation(3, 147)_ = 0.8020, P = 0.4946 and for morphine: F_analgesia(3, 38)_ = 51.50, P < 0.0001; F_respiration(3, 112)_ = 3.875, P = 0.0112; F_constipation(3, 151)_ = 5.543, P = 0.0012). One-way ANOVA with Bonferroni *post hoc* test.
