# Supplementary material for: Phosphorylation-deficient G-protein-biased μ-opioid receptors improve analgesia and diminish tolerance but worsen opioid side effects
Source: Nat Commun. 2019 Jan 21;10:367. doi: 10.1038/s41467-018-08162-1 (PMC6341117; doi:10.1038/s41467-018-08162-1)
Supplement: Supplementary file 3 — Reporting Summary [file 41467_2018_8162_MOESM3_ESM.pdf]

nature research

Corresponding author(s): NCOMMS-18-15778A

Reporting Summary

Nature Research wishes to improve the reproducibility of the work that we publish. This form provides structure for consistency and transparency in reporting. For further information on Nature Research policies, see Authors & Referees and the Editorial Policy Checklist.

Statistical parameters

When statistical analyses are reported, confirm that the following items are present in the relevant location (e.g. figure legend, table legend, main text, or Methods section).

n/a	Confirmed	
☐	☒	The exact sample size (*n*) for each experimental group/condition, given as a discrete number and unit of measurement
☐	☒	An indication of whether measurements were taken from distinct samples or whether the same sample was measured repeatedly
☐	☒	The statistical test(s) used AND whether they are one- or two-sided<br>*Only common tests should be described solely by name; describe more complex techniques in the Methods section.*
☐	☒	A description of all covariates tested
☐	☒	A description of any assumptions or corrections, such as tests of normality and adjustment for multiple comparisons
☐	☒	A full description of the statistics including central tendency (e.g. means) or other basic estimates (e.g. regression coefficient) AND variation (e.g. standard deviation) or associated estimates of uncertainty (e.g. confidence intervals)
☐	☒	For null hypothesis testing, the test statistic (e.g. *F*, *t*, *r*) with confidence intervals, effect sizes, degrees of freedom and *P* value noted<br>*Give P values as exact values whenever suitable.*
☒	☐	For Bayesian analysis, information on the choice of priors and Markov chain Monte Carlo settings
☒	☐	For hierarchical and complex designs, identification of the appropriate level for tests and full reporting of outcomes
☐	☒	Estimates of effect sizes (e.g. Cohen's *d*, Pearson's *r*), indicating how they were calculated
☐	☒	Clearly defined error bars<br>*State explicitly what error bars represent (e.g. SD, SE, CI)*

Our web collection on statistics for biologists may be useful.

Software and code

Policy information about availability of computer code

Data collection	Adobe: Illustrator, InDesign, Photoshop CS6; Microsoft Office Version 16.16
Data analysis	GraphPad Prism 6 software

For manuscripts utilizing custom algorithms or software that are central to the research but not yet described in published literature, software must be made available to editors/reviewers upon request. We strongly encourage code deposition in a community repository (e.g. GitHub). See the Nature Research guidelines for submitting code & software for further information.

Data

Policy information about availability of data

All manuscripts must include a data availability statement. This statement should provide the following information, where applicable:

- Accession codes, unique identifiers, or web links for publicly available datasets
- A list of figures that have associated raw data
- A description of any restrictions on data availability

The authors declare that all data supporting the findings of this study are available within the paper and its supplementary information files. The source data underlying all figures are provided as a Source Data file. The data that support the findings of this study are available from the authors upon reasonable request.

Field-specific reporting

Please select the best fit for your research. If you are not sure, read the appropriate sections before making your selection.

☒ Life sciences ☐ Behavioural & social sciences ☐ Ecological, evolutionary & environmental sciences

For a reference copy of the document with all sections, see nature.com/authors/policies/ReportingSummary-flat.pdf

Life sciences study design

All studies must disclose on these points even when the disclosure is negative.

Sample size	To increase statistical power, we used 6–16 mice per genotype for each behavioural experiment group.
Data exclusions	Mice were excluded from the study if they displayed any bodily injuries from aggressions with cage mates.
Replication	To increase statistical power, we used 6–16 mice per genotype for each behavioural experiment group
Randomization	Animals were assigned to groups randomly before testing.
Blinding	The experimenter was blinded to treatment and/or genotype throughout the course of behavioural testing. All drugs were given to the experimenter in coded vials and decoded after completion of the experiment.

Reporting for specific materials, systems and methods

Materials & experimental systems

n/a	Involved in the study	
☒	☐	Unique biological materials
☐	☒	Antibodies
☒	☐	Eukaryotic cell lines
☒	☐	Palaeontology
☐	☒	Animals and other organisms
☒	☐	Human research participants

Methods

n/a	Involved in the study	
☒	☐	ChIP-seq
☒	☐	Flow cytometry
☒	☐	MRI-based neuroimaging

Antibodies

Antibodies used	anti-beta-arrestin1 (ab32095, abcam), anti-GRK2 (sc-562, Santa Cruz Biotechnology), anti-actin (sc-47778, Santa Cruz Biotechnology)
Validation	see webside

Animals and other organisms

Policy information about studies involving animals; ARRIVE guidelines recommended for reporting animal research

Laboratory animals	Mice: 10S/T-A (Oprm1tm2.1Shlz, MGI:6117668), 11S/T-A (Oprm1tm3.1Shlz, MGI:6117673), S375A (Oprm1tm1Shlz), WT JAXTM C57BL/6J mice from Charles River Laboratories (DE) MGI:5000465)<br>In all behavioural experiments, we used male mice aged 8–16 weeks and weighing 25–30 g.
Wild animals	WT JAXTM C57BL/6J mice from Charles River Laboratories (DE) MGI:5000465)<br>In all behavioural experiments, we used male mice aged 8–16 weeks and weighing 25–30 g.
Field-collected samples	*For laboratory work with field-collected samples, describe all relevant parameters such as housing, maintenance, temperature, photoperiod and end-of-experiment protocol OR state that the study did not involve samples collected from the field.*
